# Rapid, ultrasensitive, and highly specific identification of *Brucella abortus* utilizing multiple cross displacement amplification combined with a gold nanoparticles-based lateral flow biosensor

**DOI:** 10.3389/fmicb.2022.1071928

**Published:** 2022-11-29

**Authors:** Xinggui Yang, Yue Wang, Ying Liu, Junfei Huang, Xiaoyu Wei, Qinqin Tan, Xiaoyan Zeng, Xia Ying, Shijun Li

**Affiliations:** ^1^Laboratory of Infectious Disease of Experimental Center, Guizhou Provincial Center for Disease Control and Prevention, Guiyang, Guizhou, China; ^2^The Second Affiliated Hospital, Guizhou University of Traditional Chinese Medicine, Guiyang, Guizhou, China

**Keywords:** *Brucella abortus*, *Bovine brucellosis*, multiple cross displacement amplification, gold nanoparticles-based lateral flow biosensor, BruAb2_0168 gene

## Abstract

*Brucella abortus* (*B. abortus*) as an important infectious agent of bovine brucellosis cannot be ignored, especially in countries/regions dominated by animal husbandry. Thus, the development of an ultrasensitive and highly specific identification technique is an ideal strategy to control the transmission of bovine brucellosis. In this report, a novel detection protocol, which utilizes multiple cross displacement amplification (MCDA) combined with a gold nanoparticles-based lateral flow biosensor (AuNPs-LFB) targeting the *BruAb2_0168* gene was successfully devised and established for the identification of *B. abortus* (termed *B. abortus*-MCDA-LFB). Ten specific primers containing engineered C1-FAM (carboxyfluorescein) and D1-biotin primers were designed according to the MCDA reaction mechanism. These genomic DNA extracted from various bacterial strains and whole blood samples were used to optimize and evaluate the *B. abortus*-MCDA-LFB assay. As a result, the optimal reaction conditions for the *B. abortus*-MCDA-LFB assay were 66°C for 40 min. The limit of detection of the *B. abortus*-MCDA-LFB was 10 fg/μl (~3 copies/μl) for genomic DNA extracted from pure cultures of *B. abortus* isolate. Meanwhile, the *B. abortus*-MCDA-LFB assay accurately identified all tested *B. abortus* strains, and there was no cross-reaction with non-*B. abortus* pathogens. Moreover, the detection workflow of the *B. abortus*-MCDA-LFB assay for whole blood samples can be completed within 70 min, and the cost of a single test is approximately 5.0 USD. Taken together, the *B. abortus*-MCDA-LFB assay is a visual, fast, ultrasensitive, low-cost, easy-to-operate, and highly specific detection method, which can be used as a rapid identification tool for *B. abortus* infections.

## Introduction

Brucellosis is an important worldwide zoonotic disease caused by members of the genus *Brucella*, and its species mainly involve *Brucella abortus* (*B. abortus*), *Brucella melitensis*, *Brucella suis*, and *Brucella canis* (Sergueev et al., 2017; Tekle et al., 2019). Globally, approximately 500,000 new cases of human brucellosis are reported annually, and infected animals are usually an important source of infection (Hinić et al., 2008; Moeini-Zanjani et al., 2020). Bovine brucellosis, mainly caused by *B. abortus*, is characterized by diseases associated with the reproductive system (including abortion, infertility and weak offspring in females, and orchitis and epididymitis in males) and poses a serious health challenge to the cattle herd (Ashmi et al., 2021; Aliyev et al., 2022). Importantly, humans are more likely to be infected by handling the feces or tissues of infected animals and even by drinking milk that is not fully pasteurized (Ashmi et al., 2021; Aliyev et al., 2022). In addition, there is currently no specific vaccine for humans against infection with *Brucella* spp. (Boggiatto et al., 2019). Thus, early, rapid, sensitive, and reliable identification of *B. abortus* is one of the ideal strategies to control the transmission of bovine brucellosis, especially in regions dominated by animal husbandry (Yagupsky et al., 2020).

Although many diagnostic techniques were established in the past, the bacteriological-based culture is still the preferred method for the detection and identification of *Brucella* pathogens (Yagupsky et al., 2020; Ashmi et al., 2021). However, the culture method undeniably has certain advantages (e.g., reliability and intuitiveness), but it is time-consuming (usually 2 to 3 days), complicated workflow of detection, and a risk of infection for laboratory personnel (Sergueev et al., 2017; Li et al., 2019c). The serological assays including Rose Bengal plate test and standard tube agglutination test are often used to screen for brucellosis, however, their specificity is indeed the major limitation. That is, the antibodies used may cross-react with other Gram-negative bacteria like *Yersinia enterocolitica* serotype O:9, *Vibrio cholerae*, and *Francisella* spp. (Sergueev et al., 2017). Hence, simple, rapid, sensitive, reliable, and readily available detection techniques are required to detect *B. abortus* in cattle and rapidly control the bovine brucellosis epidemic.

With the progress of molecular diagnosis, various molecular detection techniques, including polymerase chain reaction (PCR) and PCR-based assays have been designed and applied to the detection of *Brucella* organisms (e.g., multiplex PCR and real-time fluorescence PCR; Rees et al., 2009; Alamian et al., 2019). The PCR and PCR-based techniques, especially real-time fluorescence PCR, as a landmark nucleic acid detection method has been proven to be effective and reliable in many molecular analysis fields (Hinić et al., 2008; Surucuoglu et al., 2009). Here, the special requirements for the thermal cycler may limit its application in the field or basic laboratory, and the detection sensitivity still needs to be further improved in individuals with low bacterial load (Surucuoglu et al., 2009; Li et al., 2019b). As mentioned above, the isothermal amplification techniques, including recombinase polymerase amplification (RPA), loop-mediated isothermal amplification (LAMP), and multiple cross displacement amplification (MCDA), seem to be able to overcome the shortcomings of the PCR-based techniques, since its reaction process requires only isothermal incubation condition (Karthik et al., 2014; Li et al., 2019c; Gumaa et al., 2020). In particular, MCDA technique, as an attractive nucleic acid analysis tool, has been widely performed in the detection of a variety of pathogens, including bacteria, viruses, and fungi (Wang et al., 2015, 2021; Li et al., 2020).

Currently, the verification of isothermal amplicons mainly relies on agarose gel electrophoresis and real-time turbidimeter, which makes the detection process more complicated (Wang et al., 2015). In addition, visual indicators, including hydroxy naphthol blue (HNB), SYBR Green, and malachite green (MG), have been used to validate these amplicons in past studies, but they are difficult to distinguish specific from non-specific amplification accurately (Wang et al., 2016a, 2017). Thus, developing a visual, low-cost, and easy-to-use validation tool is essential. The gold nanoparticles-based lateral flow biosensor (AuNPs-LFB), as a visual, sensitive, reliable, and easy-to-prepare verification approach, has been designed and applied successfully to overcome the limitations mentioned above (Wang et al., 2018). As a result, the MCDA technique combined with an AuNPs-LFB biosensor (MCDA-LFB) has been performed to detect pathogens like *Leptospira-*and *SARS-CoV-2*-MCDA-LFB (Li et al., 2019a, 2020). Moreover, the *BruAb2_0168* gene, as a specific molecular target of *B. abortus* commonly used, has shown extreme stability in practical detection, providing an effective means for accurate diagnosis of bovine brucellosis (Karthik et al., 2014; Kang et al., 2015; Ashmi et al., 2021; Yang et al., 2021).

In the current study, novel multiple cross displacement amplification coupled with an engineered AuNPs-LFB biosensor targeting the *BruAb2_0168* gene was established and applied for visual, rapid, ultrasensitive, and highly specific identification of *B. abortus* (termed *B. abortus*-MCDA-LFB). In the detection workflow, exponentially amplified MCDA amplicons with special modifications (namely, FAM and biotin) can be validated in approximately 2–5 min using the AuNPs-LFB biosensor. Moreover, these performance indicators of *B. abortus*-MCDA-LFB assay including optimal reaction conditions, sensitivity, specificity, and applicability were evaluated using pure cultures and whole blood specimens.

## Materials and methods

### Ethical statement

The study was approved by the Human Ethics Committee of the Guizhou Provincial Center for Disease Control and Prevention (No. G2022-1) and complied with the Declaration of Helsinki. All data/isolates were analyzed anonymously.

### Reagents and apparatus

Bacterial genomic DNA extraction kits were obtained from Xi’an Tianlong Technology Co., Ltd. (Xi’an, China). Deoxyribonucleic acid isothermal amplification kits and MG chromogenic reagents were purchased from Tian-Jin Huidexin Technology Development Co., Ltd. (Tianjin, China). Backing card, sample pad, conjugate pad, nitrocellulose membrane, and absorbent pad were obtained from Jie-Yi Biotechnology. Co., Ltd. (Shanghai, China). Biotinylated bovine serum albumin (biotin-BSA) and rabbit anti-fluorescein antibody (anti-FITC) were purchased from Abcam. Co., Ltd. (Shanghai, China). Dye (crimson red) streptavidin-coated gold nanoparticles (SA-AuNPs) were provided by Bangs Laboratories, INC. (Indiana, USA). Real-time turbidimeter (*LA*-500) was provided by Eiken chemical Co., Ltd. (Japan). The ChemiDoc MP imaging system obtained from Bio-Rad (USA).

### Design and modification of MCDA primers

A total of 10 specific MCDA primers targeting the *BruAb2_0168* gene, including displacement primers (F1 and F2), amplification primers (D1, D2, C1, C2, R1, and R2), and cross primers (CP1 and CP2), were designed using the Primer Premier software (5.0). Among them, the engineered C1* and D1* primers were modified with carboxyfluorescein (FAM) and biotin at the 5′ end, respectively. Multiple sets of designed primers (more than 40 sets) were homologically aligned and screened using basic local alignment search tool software (BLAST). Details of the MCDA primers used in the current study (including primer sequence, modification, and length) were shown in Table 1. All primers with HPLC purification grade were synthesized by Tianyi-Huiyuan Biotech Co., Ltd. (Beijing, China).

**Table 1:** The primers used in the *B. abortus*-LAMP-LFB assay.

Primer	Sequence (5′-3′)^a^	Length^b^
*Bru*-F1	5′- GGGCCTTTATCACCTGTTC-3′	19 nt
*Bru*-F2	5′-GTTGAAGTTGCCCGTAGC-3′	18 nt
*Bru*-C1*	5′-FAM-TGATATACACCTTGTCCACGCTCAC-3′	25 nt
*Bru*-C2	5′-CCACTCTTAATTACTGGAACGGCA-3′	24 nt
*Bru*-CP1	5′-TGATATACACCTTGTCCACGCTCACCTCGGGTAGCGGGCTTA-3′	42 nt
*Bru*-CP2	5′-CCACTCTTAATTACTGGAACGGCAGCTGTCCAGGTGCCATTG-3′	42 nt
*Bru*-D1*	5′-Biotin-GCAGCGATCCAAGCG-3′	15 nt
*Bru*-D2	5′-CGATGGTCTGTTGCATG-3′	17 nt
*Bru*-R1	5′-CGTTGACCTGCTGGTT-3′	16 nt
*Bru*-R2	5′-TGTCGTCAATACTACTGG-3′	18 nt

a FAM, 6-Carboxyfluorescein.

b nt, nucleotide; mer, monomeric unit.

### Preparation of AuNPs-LFB biosensor

In the current study, the AuNPs-LFB biosensor (size 60 × 4 mm) was designed and constructed according to the reaction mechanism of MCDA with labeled primers (Wang et al., 2016b). In brief, the biotin-BSA (2.5 mg/ml) and anti-FITC (0.15 mg/ml) conjugates were immobilized on the nitrocellulose membrane to form the control line (CL, conjugating with biotin-BSA) and test line (TL, conjugating with anti-FITC). The two bands were separated by approximately 5 mm. Then, the sample pad, conjugate pad, conjugates-embedded nitrocellulose membrane, and absorbent pad were fixed to the backing card with plastic adhesive. The SA-AuNPs (129 nm, 10 mg ml^−1^, 100 mM borate, pH 8.5 with 0.1% BSA, 0.05% Tween 20, and 10 mM EDTA) were collected in the conjugate pad. The AuNPs-LFB biosensors designed in the current study were assembled by Tian-Jin HuiDeXin Biotech. Co., Ltd. (Tianjin, China). These manufactured AuNPs-LFB strips were packaged in a plastic box containing the desiccant gel and subsequently stored at room temperature in a dark and dry environment until use. After an aliquot (0.8–1.2 μl) of MCDA amplicon and two drops (100–150 μl) of reaction buffer were successively added to the sample pad of the biosensor, the results were determined by observing the TL and CL lines (approximately 2–5 min).

### Bacterial strains

A total of 28 bacterial strains, including *B. abortus* strains (reference strain 544, vaccine strain A19, and *B. abortus* isolates), *Brucella melitensis* strains (reference strain 16 M, vaccine strain M5, and *Brucella melitensis* isolates), *Brucella suis* strains (reference strain 1330S, vaccine strain S2), *Brucella canis* strain, and non-*Brucella* strains, were used to evaluate the specificity of *B. abortus*-MCDA-LFB assay. Details of these bacterial strains (including name/variant, source, number, and test results) used in the current study were displayed in Table 2. The genomic DNA of all strains examined in the current study was prepared using the universal bacterial nucleic acid extraction kits, and the extraction steps were performed according to the manufacturer’s instruction. The genomic DNA extracted from *B. abortus* isolate was quantified at the 260/280 wavelengths using an ultraviolet spectrophotometer (Thermo Fisher Scientific Co., Ltd. Beijing, China) and was prepared as serial diluents (namely, 1 ng/μl, 100 pg/μl, 10 pg/μl, 1 pg/μl, 100 fg/μl, 10 fg/μl, and 1 fg/μl). Moreover, the DNA copy number was calculated using a conventional formula. The DNA copy number (copy number/μl) = [6.02 × 10^23^ × genomic DNA concentration (ng/μl) × 10^−9^]/[genomic DNA length (nt) × 660] (Li et al., 2022).

**Table 2:** The information of bacterial strains in this study.

Bacteria	Strain no. (source of strains)^a^	No. of strains	MCDA-LFB result^b^
*Brucella* species			
*B. abortus*	544 (ATCC 23448)	1	P
*B. abortus*	A19 (GZCDC)	1	P
*B. abortus*	Isolated strain (GZCDC)	3	P
*B. melitensis*	16 M (ATCC 23456)	1	N
*B. melitensis*	M5 (GZCDC)	1	N
*B. melitensis*	Isolated strains (GZCDC)	6	N
*B. suis*	1330S (ATCC 23444)	1	N
*B. suis*	S2 (GZCDC)	1	N
*B. canis*	Isolated strain (GZCDC)	1	N
Non-*Brucella* species			
*Mycobacterium tuberculosis*	H37Rv (ATCC 27294)	1	N
*Mycobacterium bovis*	ATCC 19210	1	N
*Listeria monocytogenes*	Isolated strain (GZCDC)	1	N
*Klebsiella pneumoniae*	Isolated strain (GZCDC)	1	N
*Streptococcus pneumoniae*	Isolated strain (GZCDC)	1	N
*Pseudomonas aeruginosa*	Isolated strains (GZCDC)	1	N
*Haemophilus influenzae*	Isolated strains (GZCDC)	1	N
*Staphylococcus aureus*	Isolated strains (GZCDC)	1	N
*Shigella sonnei*	Isolated strains (GZCDC)	1	N
*Bacillus anthracis*	Isolated strains (GZCDC)	1	N
*Streptococcus suis*	Isolated strains (GZCDC)	1	N
*Salmonella* spp.	Isolated strains (GZCDC)	1	N
Total		28	

a ATCC, American Type Culture Collection; GZCDC, Guizhou Provincial Center for Disease Control and Prevention.

b P, positive; N, negative.

### Processing of whole blood samples

A total of 56 whole blood samples of suspected bovine brucellosis collected from different regions of Guizhou Province were divided into two equal parts (Part I and Part II), which were subjected to traditional culture method and molecular detection (*B. abortus*-PCR, *B. abortus*-LAMP-LFIA and *B. abortus*-MCDA-LFB). Part I (approximately 3 ml) was aseptically injected into a two-phase culture flask (BIOVD, Zhengzhou, Henan, China) and subsequently incubated at 37°C and 5% CO_2_ atmosphere for 3–5 days (blind passage for more 3–5 days of cultivation may be needed) for *Brucella* culture and isolation (Li et al., 2019c). After all primary culture-positive strains were inoculated on both a blood agar plate and a *Brucella* agar plate, the colony morphology and characteristics (e.g., smooth and rough colonies) were observed using an inverted microscope. Then, the conventional biochemical tests (including Gram staining, CO_2_ requirements, H_2_S production, agglutination with monospecific antisera, phage lysis test) were performed to further identify *B. abortus* in our current study. If no colonies were observed, the culture flask was tilted and re-incubated for at least 30 days, and the culture was processed as the primary culture-positive strain if colony growth was observed during the culture period. If still no growth was observed after 30 days, the blood sample judged as culture-negative (Barua et al., 2016). In addition, another part of whole blood samples (Part II) was used to prepare DNA templates utilizing the protocol of QIAamp (Li et al., 2019b).

### The *Brucella abortus*-MCDA-LFB reaction

The 25 microliters of MCDA reaction mixture consisted of the following: 12.5 μl reaction buffer (2 ×), 1 μl *Bst* DNA polymerase (8 U), 0.4 μM each of displacement primer (*Bru*-F1 and *Bru-F2*), 0.8 μM each of amplification primer (*Bru*-C1*, *Bru*-C2, *Bru*-R1, *Bru*-R2, *Bru*-D1, and *Bru*-D2*), 1.6 μM each of cross primer (*Bru*-CP1 and *Bru*-CP2), 1 μl visual reagents (MG), 1 μl of genomic DNA template extracted from pure cultures or 5 μl of DNA templates from whole blood specimens, and then nuclease-free water was added to 25 μl. The amplification system was incubated at 63°C for 60 min and then inactivated at 85°C for 5 min. In the current study, negative controls (NC) included 1 μl of genomic DNA from *Mycobacterium tuberculosis* (H37Rv) and 1 μl of environmental sample in the experiment, while 1 μl of nuclease-free water served as a blank control (BC). Then, the MCDA amplicons were detected using the AuNPs-LFB biosensor, MG visual reagents, real-time turbidimeter, or 1.5% agarose gel electrophoresis.

### Optimization of *Brucella abortus*-MCDA-LFB amplification conditions

In order to obtain the optimal detection efficiency, the optimization tests of temperature for *B. abortus*-MCDA-LFB assay were performed at different amplification temperatures (ranging from 60 to 67°C with 1°C intervals). One microliter of genomic DNA extracted from the isolate (1 pg/μl) was used as an amplification template. According to the *B. abortus*-MCDA-LFB reaction system, the amplicons were monitored using a real-time turbidimeter when the *B. abortus*-MCDA reaction was completed. In the current study, the turbidity threshold value was automatically set to 0.1, and a turbidity value >0.1 was judged as positive amplification (<0.1 was negative). In addition, the optimal reaction time of *B. abortus*-MCDA-LFB was also optimized to achieve the purpose of rapid detection of *B. abortus* pathogens. Serial dilutions of genomic DNA from *B. abortus* isolate (i.e., 1 ng/μl, 100 pg/μl, 10 pg/μl, 1 pg/μl, 100 fg/μl, 10 fg/μl, and 1 fg/μl) were examined by setting different amplification times (ranging from 10 to 60 min with 10 min intervals) with reference to the *B. abortus*-MCDA-LFB reaction system. Then, 1 μl of the DNA dilution mentioned above was added to the MCDA mixture, and the amplification products were verified using an AuNPs-LFB biosensor. As a result, the result was considered positive when both CL and TL regions presented red bands, but negative when only CL regions showed red band.

### Sensitivity and specificity testing of *Brucella abortus*-MCDA-LFB assay

To explore the detection sensitivity of the *B. abortus*-MCDA-LFB assay, 1 μl of each diluent of genomic DNA (i.e., 1 ng/μl, 100 pg/μl, 10 pg/μl, 1 pg/μl, 100 fg/μl, 10 fg/μl, and 1 fg/μl) was detected according to the *B. abortus*-MCDA reaction system and optimal amplification conditions (66°C, 40 min). Amplicons of *B. abortus*-MCDA were validated using AuNPs-LFB biosensor, MG amplification indicator, 1.5% agarose gel electrophoresis, and real-time turbidimeter. The limit of detection (LoD) of the *B. abortus*-MCDA-LFB assay was defined as the lowest concentration of genomic DNA that can detect *B. abortus* in ≥95% of the tests performed in the current study when the serial dilutions were tested in duplicate (usually 20 times; Chakravorty et al., 2017).

Moreover, the detection sensitivity of the *B. abortus*-MCDA-LFB assay was also analyzed using genomic DNA extracted from various pathogens, including five strains of *B. abortus*, eight strains of *Brucella melitensis*, two strains of *Brucella suis*, one strain of *Brucella canis*, and 12 strains of non-*Brucella* strains. One microliter of genomic DNA from each bacterial strain was added to the *B. abortus*-MCDA mixture system. The specificity test of *B. abortus*-MCDA-LFB assay was carried out according to the optimal amplification conditions. Follow-up, these results were reported using an AuNPs-LFB biosensor.

### Evaluation of the *Brucella abortus*-MCDA-LFB assay for detection of whole blood samples

Fifty-six whole blood samples were used to evaluate the applicability of *B. abortus*-MCDA-LFB assay by comparing with conventional culture and *B. abortus*-LAMP-LFIA methods. The detection workflow of the culture method is as mentioned above. Five microliters of genomic DNA extracted from whole blood samples were used as a template in the *B. abortus*-PCR/ -MCDA-LFB/ -LAMP-LFIA assays. The results of the traditional culture assay were used as a standard control for further analysis with *B. abortus*-PCR, *B. abortus*-LAMP-LFIA, and *B. abortus*-MCDA-LFB assays. The *B. abortus*-MCDA-LFB assay was implemented according to the reaction system and optimal reaction conditions mentioned above, and the results were monitored using the AuNPs-LFB biosensor. The *B. abortus*-LAMP-LFIA and-PCR assays were conducted according to the previous publications (Hinić et al., 2008; Yang et al., 2021), and the experimental details were supplemented in the Supplementary material.

## Results

### An overview of the *Brucella abortus*-MCDA-LFB assay

**Figure 1:**
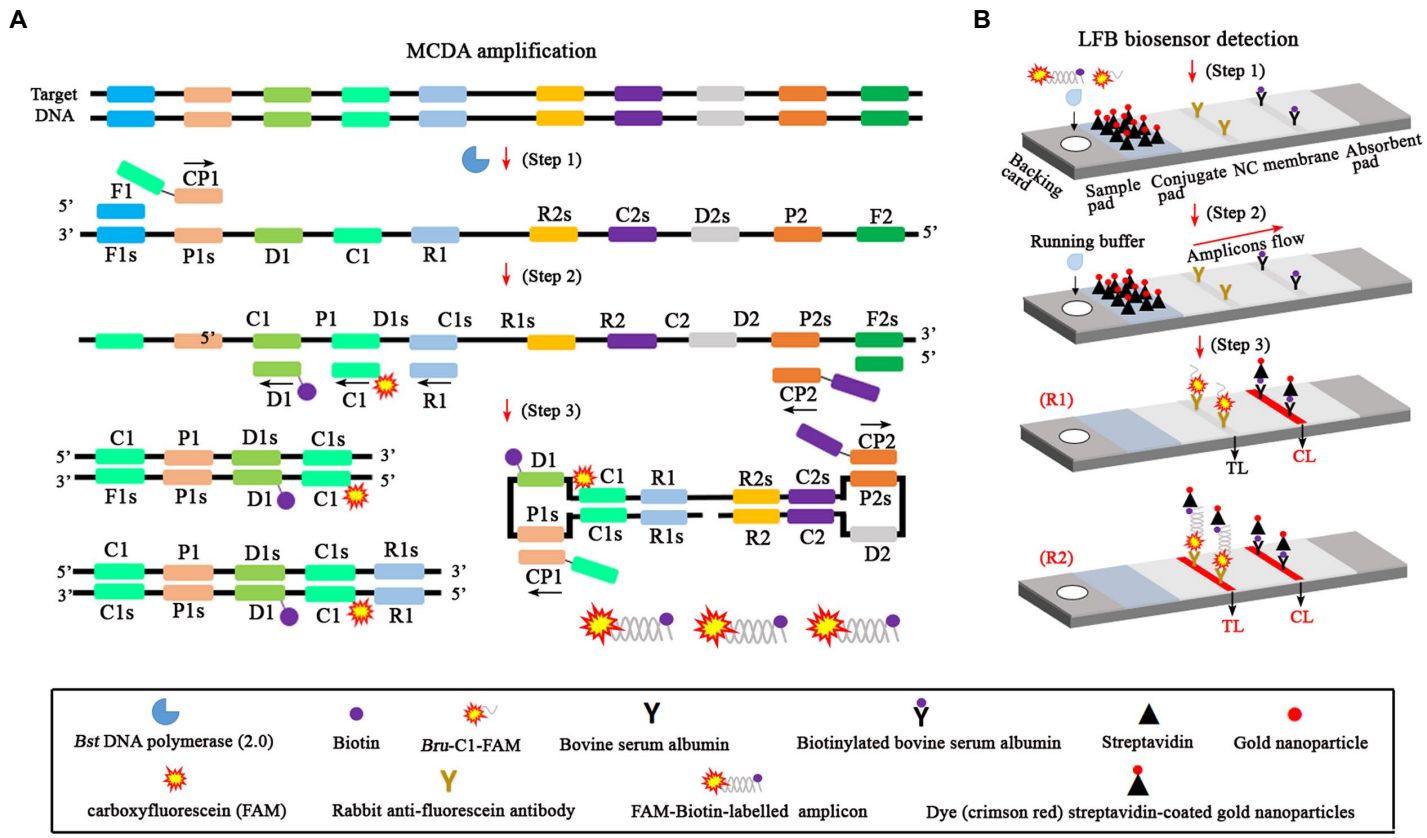
An overview of the detection principle of the *Brucella abortus*-MCDA-LFB assay. The whole process of the *B. abortus*-MCDA-LFB assay included MCDA amplification **(A)** and AuNPs-LFB biosensor detection **(B)**. In this report, the DNA templates were prepared from whole blood samples (**A**, **step 1**), and the target regions on the template were amplified exponentially with primers driven by the *Bst* DNA polymerase (**A, steps 2 and 3**). One aliquot of MCDA amplicons with the FAM and biotin labels (0.8–1.2 μl) and two drops of running buffer (100–150 μl) was added to the sample pad on the AuNPs-LFB biosensor (**B, step 1**). Subsequently, the biotin was coupled with SA-AuNP particles in conjugated pads to form the FAM/target/biotin/SA-AuNP complexes, which were captured by an anti-FITC antibody, resulting in a red TL line (**B, steps 2 and 3**). MCDA, multiple cross displacement amplification; AuNPs-LFB, gold nanoparticles-based lateral flow biosensor; TL, test line; CL, control line; FAM, carboxyfluorescein.

**Figure 2:**
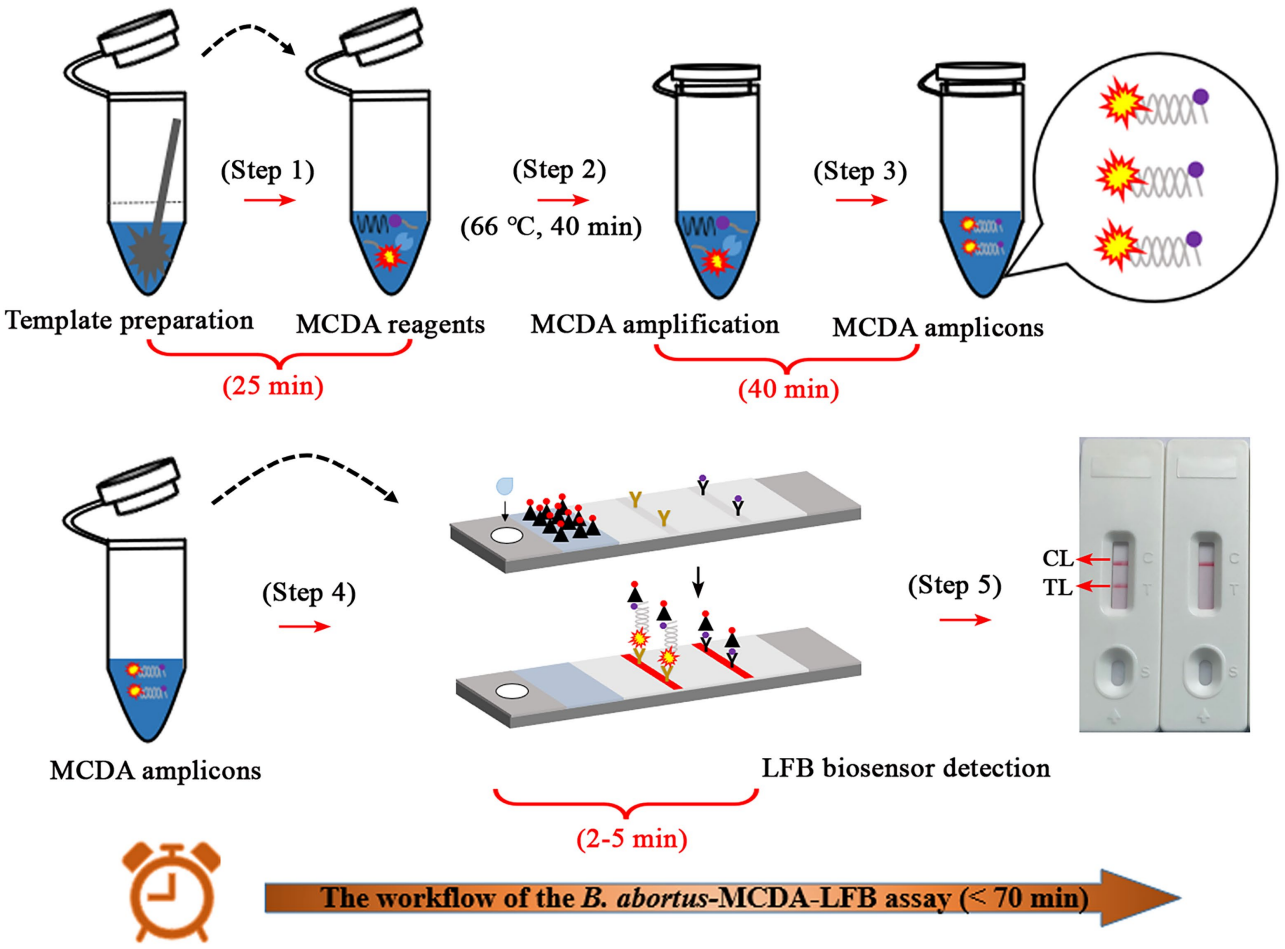
Description of the detection workflow of the *B. abortus*-MCDA-LFB assay. The detection workflow of the *B. abortus*-MCDA assay mainly consists of three steps: the DNA templates were rapidly prepared (**step 1**), the reaction tubes premixed with DNA template, amplification reagents, and MCDA primers were incubated at 66°C for 40 min (**steps 2 and 3**), and then the amplicons were verified using the AuNPs-LFB biosensor (**steps 4 and 5**). Finally, the entire workflow of the *B. abortus*-MCDA-LFB assay for whole blood samples can be completed within 70 min, including DNA template preparation (25 min), MCDA amplification (40 min), and AuNPs-LFB biosensor validation (2–5 min). MCDA, multiple cross displacement amplification; AuNPs-LFB, gold nanoparticles-based lateral flow biosensor; TL, test line; CL, control line.

The whole workflow of the *B. abortus*-MCDA-LFB assay included MCDA amplification and AuNPs-LFB biosensor detection in the current study (Figures 1, 2). Briefly, the genomic DNA prepared from whole blood samples was used as an amplification template (Figure 1A, step 1), and the target region on the template was amplified exponentially with primers driven by the *Bst* DNA polymerase (Figure 1A, steps 2 and 3). One aliquot of MCDA amplicons with the FAM and biotin labels (0.8–1.2 μl) and two drops of running buffer (100–150 μl) was added to the sample pad on the AuNPs-LFB biosensor (Figure 1B, step 1). Subsequently, the biotin was coupled with SA-AuNPs particles in conjugated pads to form the FAM/target/biotin/SA-AuNP complexes, which were captured by an anti-FITC antibody, resulting in a red TL line (Figure 1B, steps 2 and 3). As a result, the whole detection procedure of the *B. abortus*-MCDA-LFB assay for whole blood samples can be completed within 70 min, including DNA template preparation (25 min), MCDA amplification (40 min), and AuNPs-LFB biosensor validation (2–5 min; Figure 2).

### Confirmation test of *Brucella abortus*-MCDA assay

**Figure 3:**
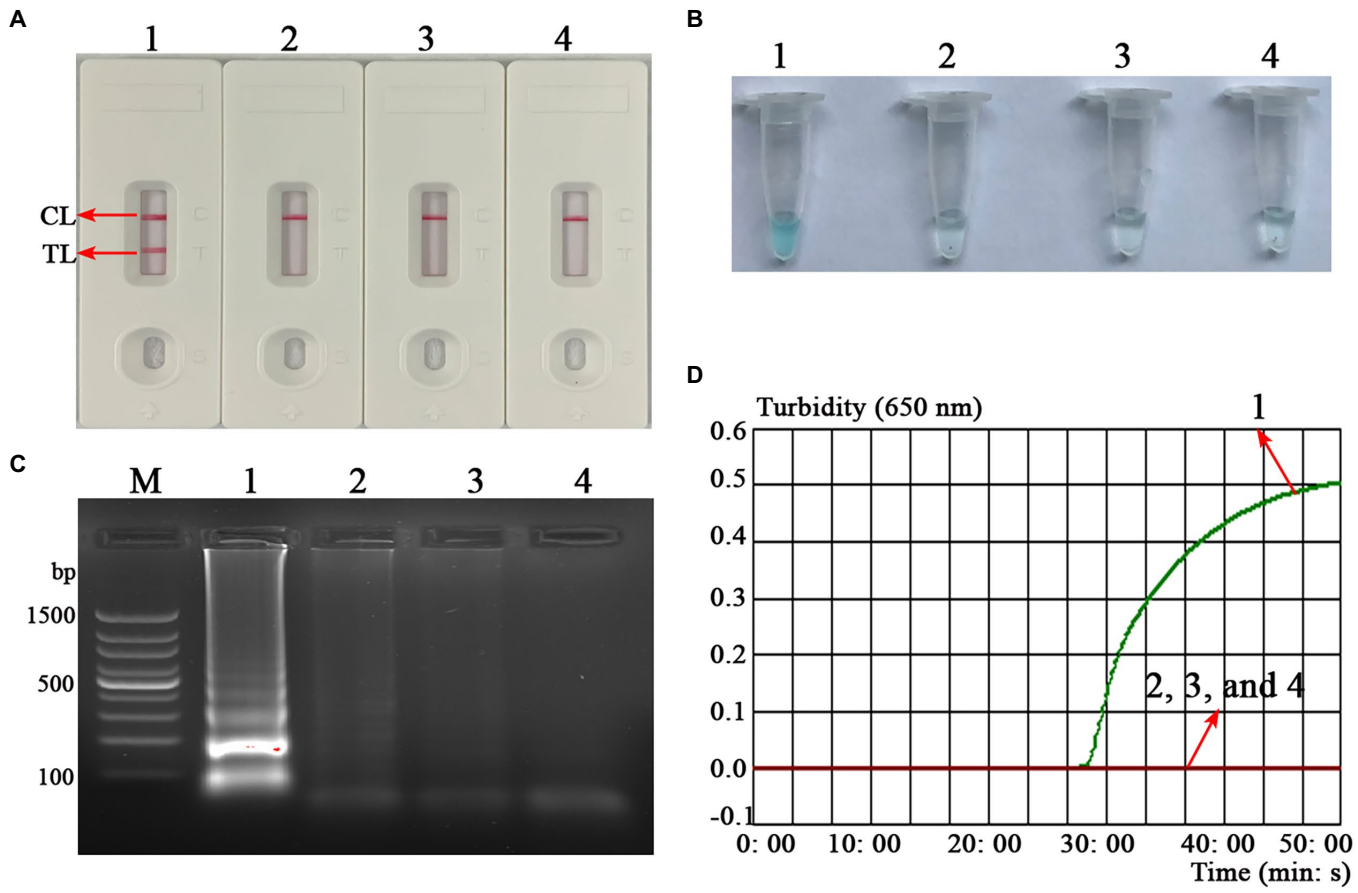
Confirmation test of *B. abortus*-MCDA-LFB assay. To verify the feasibility of the *B. abortus*-MCDA-LFB assay, the amplification products were reported using AuNPs-LFB biosensor **(A)**, MG visualization reagents **(B)**, 1.5% agarose gel electrophoresis **(C)**, and real-time turbidimetry **(D)**. Strip (**A1**)/tube (**B1**)/lane (**C1**)/curve (**D1**): positive reactions of the *B. abortus*-MCDA-LFB assay; Strip (**A2**)/tube (**B2**)/lane (**C2**)/curve (**D2**): negative control (*Mycobacterium tuberculosis*) of the *B. abortus*-MCDA-LFB assay; Strip (**A3**)/tube (**B3**)/lane (**C3**)/curve (**D3**): negative control (environmental sample in the test) of the *B. abortus*-MCDA-LFB assay; Strip (**A4**)/tube (**B4**)/lane (**C4**)/curve (**D4**): blank control (nuclease-free water) of the *B. abortus*-MCDA-LFB assay. Line **M**, 100 bp DNA ladder; MCDA, multiple cross displacement amplification; AuNPs-LFB, gold nanoparticles-based lateral flow biosensor; TL, test line; CL, control line.

To confirm the feasibility of the *B. abortus*-MCDA test, the amplification products were reported using AuNPs-LFB biosensor MG visualization reagents, 1.5% agarose gel electrophoresis, and real-time turbidimetry. Here, the TL and CL lines presented red bands on the AuNPs-LFB biosensor in positive amplification, while only CL lines were red for both negative control and blank control (Figure 3A). The positive reaction tube displayed blue, while the negative reaction tubes were light blue or colorless under the action of MG reagents (Figure 3B). The amplicon of the positive reaction was electrophoresed on 1.5% agarose gel with characteristic gradient band (Figure 3C). Subsequently, the turbidity value of positive amplification was greater than 0.1, while that of negative reaction was less than 0.1 (Figure 3D).

### Optimal reaction conditions for *Brucella abortus*-MCDA-LFB assay

**Figure 4:**
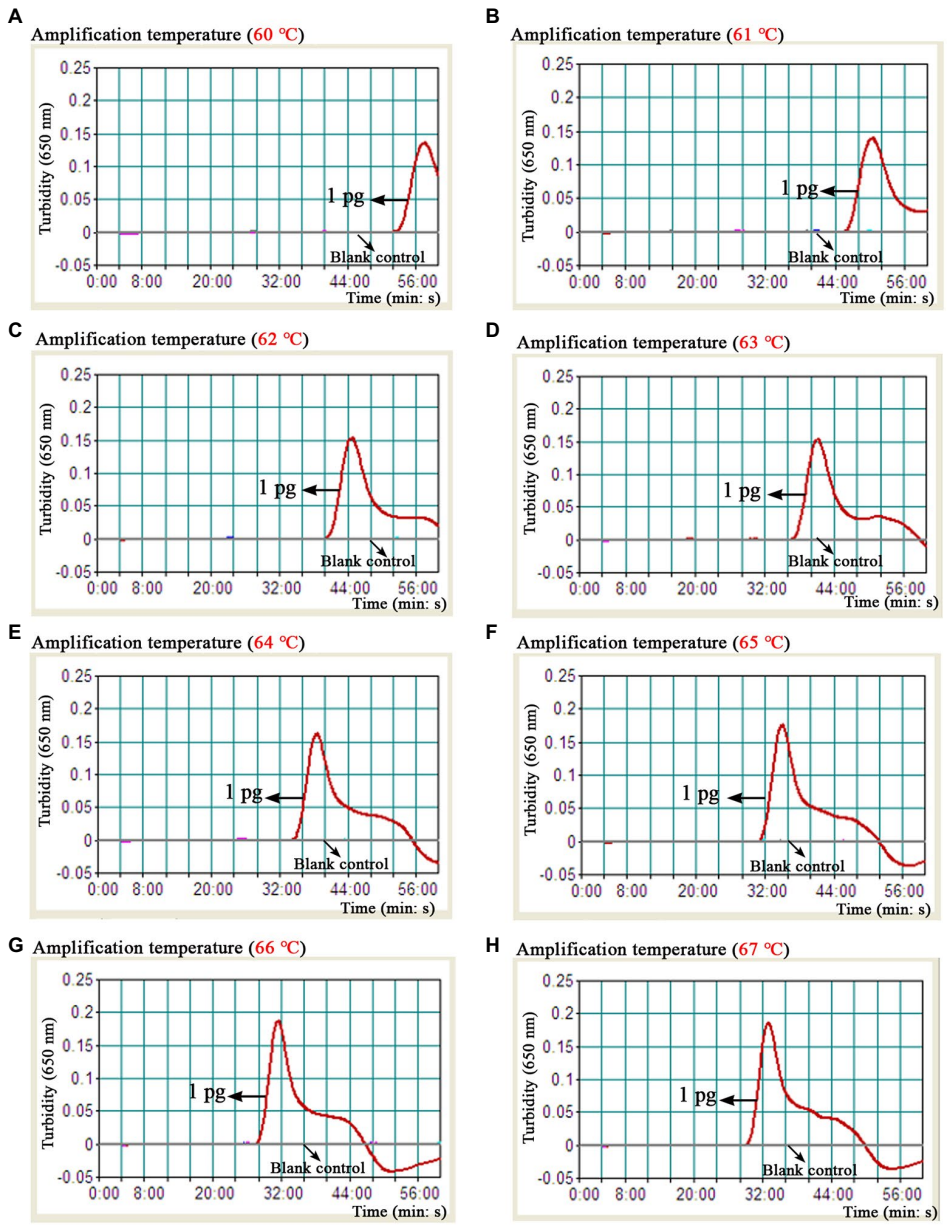
Optimal reaction temperature of the *B. abortus*-MCDA-LFB assay. The results of the *B. abortus*-MCDA-LFB assay were reported using a real-time turbidimeter, and a turbidity value greater than 0.1 was considered as positive (threshold value was 0.1). Eight dynamic curves **(A–H)** showed that the amplification efficiency of the *B. abortus*-MCDA-LFB assay was relatively high when the temperature range was 66°C to 67°C (**G,H**), and the optimal temperature was 66°C (**G**). MCDA, multiple cross displacement amplification; AuNPs-LFB, gold nanoparticles-based lateral flow biosensor; TL, test line; CL, control line.

**Figure 5:**
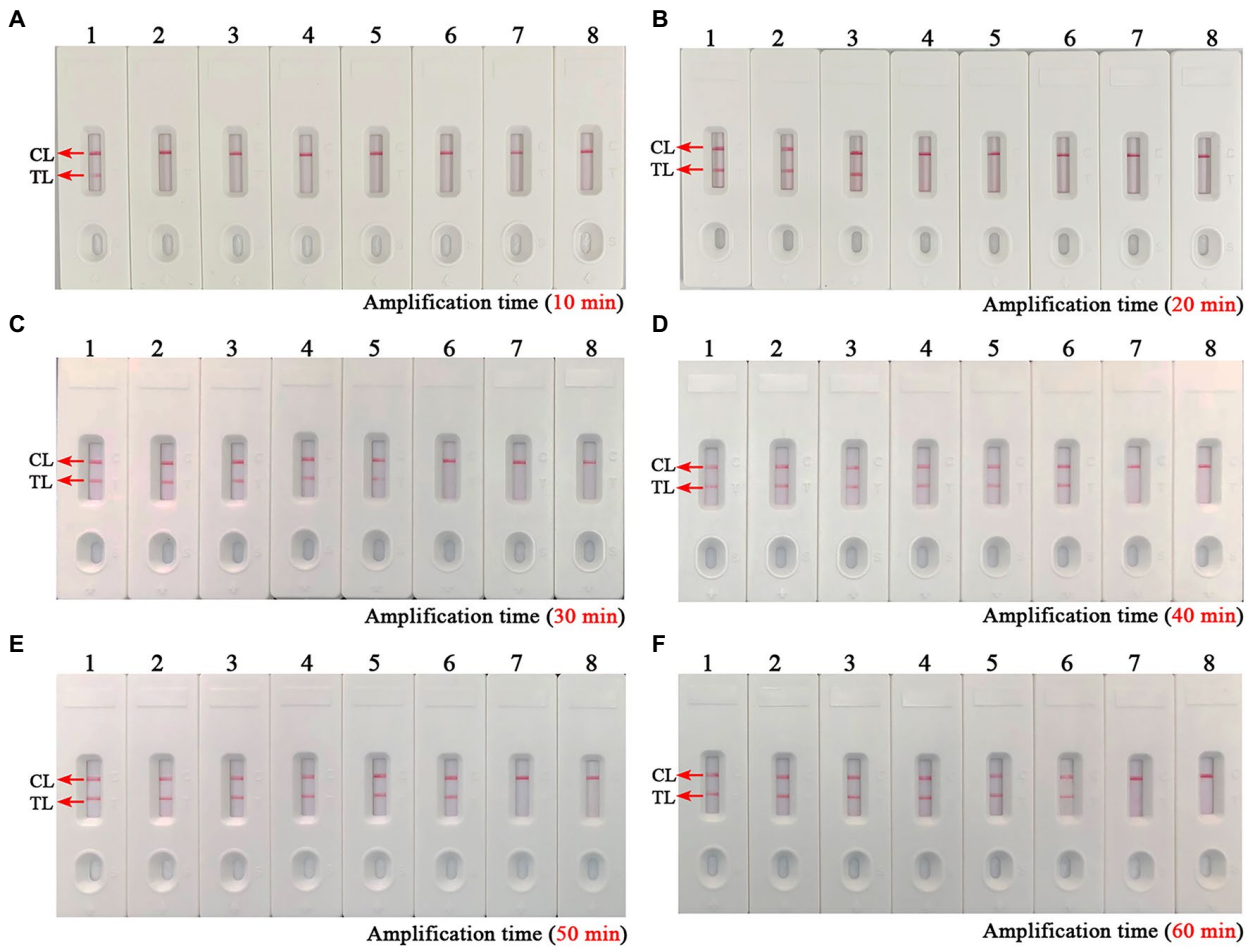
Optimal reaction time of the *B. abortus*-MCDA-LFB assay. The optimal reaction time of the *B. abortus*-MCDA-LFB assay was explored by examining each diluent of genomic DNA of the *B. abortus* strain (namely, 1 ng/μl, 100 pg/μl, 10 pg/μl, 1 pg/μl, 100 fg/μl, 10 fg/μl, and 1 fg/μl). The plots (**A–F**) correspond to 10 min, 20 min, 30 min, 40 min, 50 min, and 60 min, respectively. Biosensors 1–7 (**A–F**) correspond to 1 ng/μl, 100 pg/μl, 10 pg/μl, 1 pg/μl, 100 fg/μl, 10 fg/μl, and 1 fg/μl. Biosensors 8 (**A–F**) correspond to blank control (nuclease-free water). When the reaction time ranged from 40 to 60 min, the LoD of the *B. abortus*-MCDA-LFB assay was 10 fg/microliter. As a result, the optimal amplification time of *B. abortus*-MCDA-LFB assay was 40 min (**D**) in the current study. MCDA, multiple cross displacement amplification; AuNPs-LFB, gold nanoparticles-based lateral flow biosensor; TL, test line; CL, control line.

As shown in Figure 4, a total of eight turbidity curves (Figures 4A–H) were generated using the real-time turbidity meter in the temperature optimization test. Two kinetic plots (Figures 4G,H) showed higher amplification efficiency than the rest of curves, especially the G-plot (namely, 66°C). Thus, the 66°C was used as the optimal amplification temperature for the *B. abortus*-MCDA-LFB assay in subsequent experiments. Moreover, when the amplification time was 40–60 min, the lowest concentration of *B. abortus* genomic DNA detected by *B. abortus*-MCDA-LFB assay was 10 fg/μl in the time optimization test (Figure 5). To achieve the purpose of rapid detection, 40 min was used as the optimal amplification time of *B. abortus*-MCDA-LFB assay (Figure 5D). As a result, the optimal amplification conditions of *B. abortus*-MCDA-LFB assay were 66°C for 40 min in the current study.

### Sensitivity of the *Brucella abortus*-MCDA-LFB assay

**Figure 6:**
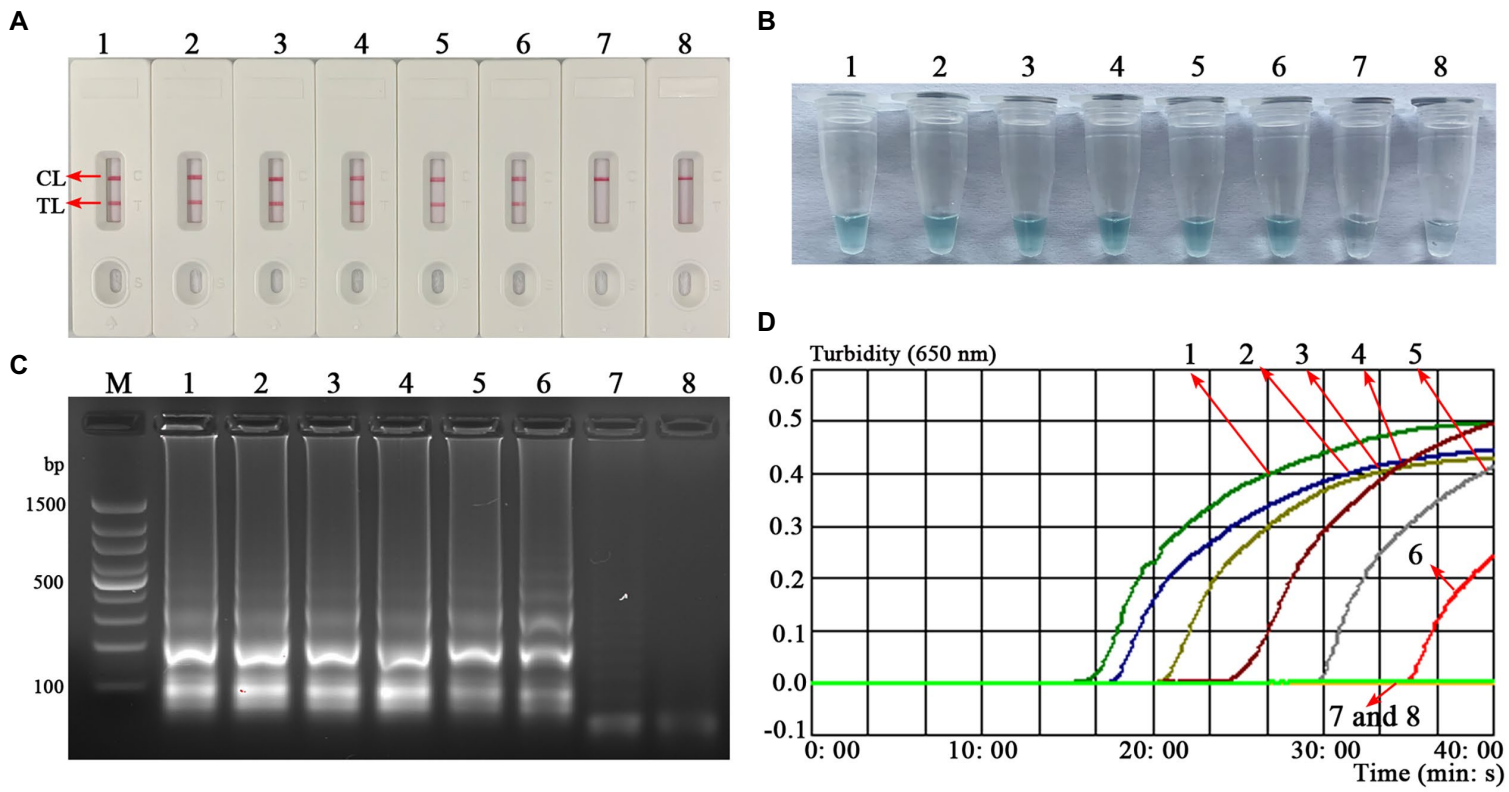
Analytical sensitivity of the *B. abortus*-MCDA-LFB assay. One microliter of each diluent of genomic DNA from the *B. abortus* strain (1 ng/μl, 100 pg/μl, 10 pg/μl, 1 pg/μl, 100 fg/μl, 10 fg/μl, and 1 fg/μl) was used as an amplification template to evaluate the analytical sensitivity of the *B. abortus*-MCDA-LFB assay. The AuNPs-LFB biosensor (**A**), MG amplification indicators (**B**), 1.5% agarose gel electrophoresis (**C**), and real-time turbidity (**D**) were used to validate the amplification products of the *B. abortus*-MCDA assay. Strip (**A1–A7**)/tube (**B1–B7**)/lane (**C1–C7**)/curve (**D1–D7**) correspond to 1 ng/μl, 100 pg/μl, 10 pg/μl, 1 pg/μl, 100 fg/μl, 10 fg/μl, and 1 fg/μl. Strip (**A8**)/tube (**B8**)/lane (**C8**)/curve (**D8**) correspond to blank control (nuclease-free water). Line **M**, 100 bp DNA ladder; MCDA, multiple cross displacement amplification; AuNPs-LFB, gold nanoparticles-based lateral flow biosensor; TL, test line; CL, control line.

The sensitivity test was performed to verify the ability of the *B. abortus*-MCDA-LFB to detect low concentrations of genomic DNA. As shown in Figure 6, the LoD of the *B. abortus*-MCDA-LFB assay established in the current study was 10 fg/μl (~3 copies/μl). Importantly, the results of the AuNPs-LFB biosensor, MG amplification reagent, 1.5% agarose gel electrophoresis, and real-time turbidimeter were consistent, confirming the reliability of the sensitivity test.

### Specificity of the *Brucella abortus*-MCDA-LFB assay

**Figure 7:**
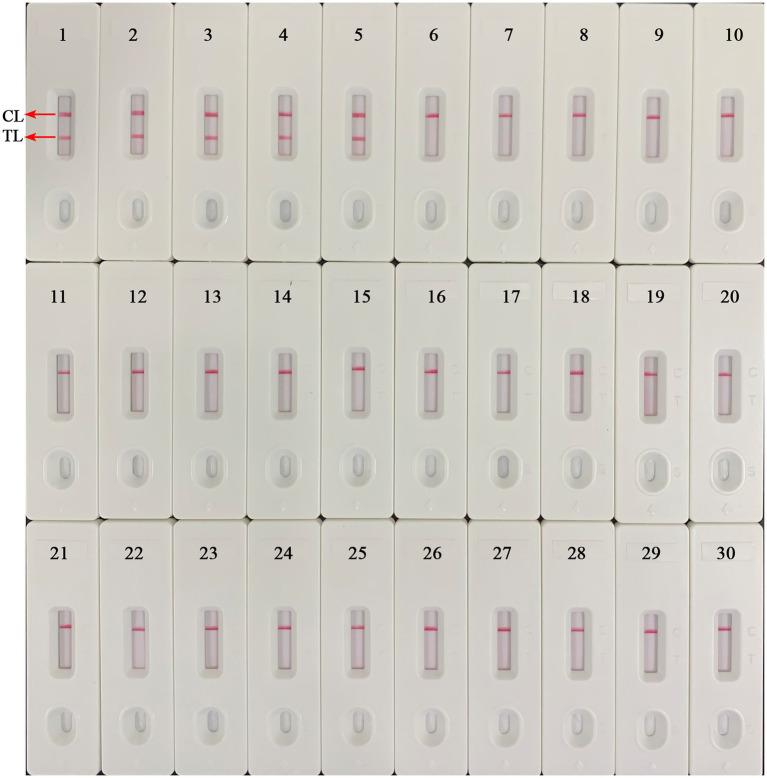
Analytical specificity of the *B. abortus*-MCDA-LFB assay. The genomic DNA extracted from 28 bacterial strains was used to evaluate the analytical specificity of the *B. abortus*-MCDA-LFB assay. Biosensor 1, *B. abortus* 544 (ATCC 23448); Biosensor 2, *B. abortus* (vaccine strain); Biosensors 3–5, *B. abortus* isolates (GZCDC); Biosensor 6, *B. melitensis* 16 M (ATCC 23456); Biosensor 7, *B. melitensis* M5 (vaccine strain); Biosensor 8–13, *B. melitensis* isolates (GZCDC); Biosensors 14, *B. suis* 1330S (ATCC 23444), Biosensors 15, *B. suis* S2 (vaccine strain); Biosensors 16, *B. canis* isolate (GZCDC); Biosensors 17–28, *Mycobacterium tuberculosis* H37Rv (ATCC 27294), *Mycobacterium bovis* (ATCC 19210), *Listeria monocytogenes* (GZCDC), *Klebsiella pneumoniae* (GZCDC), *Streptococcus pneumoniae* (GZCDC), *Pseudomonas aeruginosa* (GZCDC), *Haemophilus influenzae* (GZCDC), *Staphylococcus aureus*(GZCDC), *Shigella sonnei* (GZCDC), *Bacillus anthracis* (GZCDC), *Streptococcus suis* (GZCDC), *Salmonella* spp. (GZCDC); Biosensors 29–30, negative control (environmental sample in the test) and blank control (nuclease-free water). MCDA, multiple cross displacement amplification; AuNPs-LFB, gold nanoparticles-based lateral flow biosensor; TL, test line; CL, control line; GZCDC, Guizhou Provincial Center for Disease Control and Prevention.

The *B. abortus*-MCDA-LFB assay was able to accurately identify all *B. abortus* pathogens examined in the current study (including reference strain, vaccine strain, and isolates), and no cross-reactions occurred with other *Brucella* spp. (namely, including *Brucella melitensis*, *Brucella suis*, and *Brucella canis*) and non-*Brucella* strains (Figure 7). These data demonstrated that the specificity of MCDA-LFB assay for the identification of *B. abortus* strains was 100%.

### Practicability of *Brucella abortus*-MCDA-LFB assay for whole blood samples

To evaluate the applicability of *B. abortus*-MCDA-LFB, 56 whole blood samples collected from cattle suspected of brucellosis were subjected to conventional culture, *B. abortus*-PCR, -LAMP-LFIA, and-MCDA-LFB assays. Here, nine whole blood samples were detected as positive and 45 were negative using traditional culture, *B. abortus*-LAMP-LFIA, and *B. abortus*-MCDA-LFB assays in the current study (Table 3; Supplementary Figure S1). In the *B. abortus*-PCR assay, seven whole blood samples were tested positive and 47 were negative (Table 3; Supplementary Figure S1). Notably, the detection results of the *B. abortus*-MCDA-LFB assay were consistent (100%) with the culture assay, confirming its applicability to the detection of whole blood samples. In addition, *B. abortus*-MCDA-LFB assay (9/9) has a higher detection sensitivity for the detection of whole blood samples than the *B. abortus*-PCR test (7/9). Taken together, these data indicated that *B. abortus*-MCDA-LFB was suitable for the detection of whole blood specimens in cattle and can be used as a valuable screening/diagnostic tool for bovine brucellosis.

**Table 3:** Comparison of four methods for the detection of 56 whole blood samples.

Methods^a^	Culture	
	Positive	Negative
*B. abortus-*PCR		
Positive	7	0
Negative	2	47
		
*B. abortus*-LAMP-LFIA		
Positive	9	0
Negative	0	47
		
*B. abortus*-MCDA-LFB		
Positive	9	0
Negative	0	47

a PCR, polymerase chain reaction; LAMP, loop-mediated isothermal amplification; LFIA, lateral flow immunoassay biosensor; MCDA, multiple cross displacement amplification; AuNPs-LFB, gold nanoparticles-based lateral flow biosensor.

## Discussion

*Brucella abortus* pathogen as an important infectious agent of bovine brucellosis cannot be ignored, especially in countries/regions dominated by animal husbandry (Karthik et al., 2014; Rahdar et al., 2018; Paul et al., 2020). Although traditional culture, serology, and biochemical tests are commonly used to diagnose bovine brucellosis, these methods are time-consuming, low-sensitivity, or prone to cross-reaction with other organisms (Sergueev et al., 2017). So far, the PCR and PCR-based techniques (e.g., real-time PCR assay) have been used as a conventional method in the molecular detection of *Brucella* spp.; still, their requirement for special instruments (e.g., thermal cycler) may be a limitation in the field or resource-limited areas (Probert et al., 2004; Kang et al., 2011). Thus, the development of a rapid, ultrasensitive, easy-to-use, highly specific, and readily available detection method for *B. abortus* pathogen is an ideal strategy to prevent and control the transmission of bovine brucellosis.

Currently, isothermal-based amplification techniques like LAMP and MCDA assays own the ability to overcome the deficiencies mentioned above because they only require a thermostatic device (or even thermostatic water bath; Notomi et al., 2000; Karthik et al., 2014; Kang et al., 2015; Li et al., 2019c; Gomes et al., 2020). Notably, the MCDA technique, as an attractive protocol, has a higher sensitivity than conventional LAMP assay (typically 10-fold; Yang et al., 2021). Meanwhile, a set of MCDA primers can specifically recognize 10 different regions of the target sequence, demonstrating high specificity to the target pathogen (Wang et al., 2015). Although visual reagents (e.g., SYBR Green HNB, and MG indicators) were proven effective for the validation of MCDA amplicons, the instability resulted in the inability to accurately distinguish between specific and non-specific amplifications (Wang et al., 2018; Yang et al., 2021). Moreover, other verification methods like agarose gel electrophoresis and real-time turbidity have been successfully applied to verify the MCDA amplicons. Still, they involve the use of specialized instruments (i.e., electrophoresis apparatus and real-time turbidimeter), especially agarose gel electrophoresis with an additional electrophoresis step. Thus, the verification of MCDA amplicons requires a visual, sensitive, reliable, easy-to-operate, and readily available method.

Gold nanoparticles-based LFB biosensor, as an intuitive, reliable, low-cost, and easy-to-use verification technique, was prepared in the current study, conforming to the requirements mentioned above (Wang et al., 2018). In this report, the MCDA technique combined with an AuNPs-LFB biosensor targeting the *BruAb2_0168* gene (namely, *B. abortus*-MCDA-LFB assay) was successfully established and applied to the detection of *B. abortus* in pure cultures or whole blood samples. The detection result was determined by visualizing the CL and TL lines on the AuNPs-LFB biosensor. The strategy dramatically shortens the detection time (approximately 2–5 min) and simplifies the detection workflow (one-step method), especially without a special detector. Moreover, the AuNPs-LFB biosensor relied on the specific binding mechanism between FAM fluorescein and anti-FITC antibody (that is, the FAM/target/biotin/AuNPs complexes are captured by anti-FITC to form the TL line), demonstrating its high specificity and stability in our experiment. As expected, the assessment test confirmed that the AuNPs-LFB assay is more intuitive, convenient, and easy-to-determine than the MG reagent, 1.5% agarose gel electrophoresis, and real-time turbidity (Figure 3).

Presently, although the detection techniques based on *IS711* sequence (*IS711*-PCR, -RPA, and-LAMP assays) and *Bscp31* gene (*Bscp31*-LAMP-LFB, and-MCDA-LFB assays) were developed to detect *Brucella* spp. (genus level), they cannot accurately identify *B. abortus* (species level; Karthik et al., 2014; Batinga et al., 2018; Rahdar et al., 2018; Li et al., 2019b, 2019c). Although the multiplex PCR-based methods like AMOS-and Bruce-ladder PCR assays can identify *Brucella* species, these techniques require a long detection period (approximately 2.5 h) and are particularly associated with a complex detection workflow (typically requiring isolation and culture of *Brucella* spp.; López-Goñi et al., 2008; Eltawab et al., 2020). Thus, the *BruAb2_0168* gene, as a specific molecular target of *B. abortus*, was found in further studies, and the PCR and LAMP techniques targeting this gene were proved to be able to identify *B. abortus* pathogens in past studies (Karthik et al., 2014; Kang et al., 2015; Ashmi et al., 2021). In order to improve the detection capability (e.g., detection sensitivity, specificity, and time), the *BruAb2_0168* gene was used as a target sequence to design MCDA primers in our study, in which C1* and D1* primers were modified with FAM and biotin according to the principle of the AuNPs-LFB biosensor. In this report, the optimization test was performed to explore the optimal reaction temperature for the *B. abortus*-MCDA-LFB assay, and the results showed that amplification efficiency was higher at 66–67°C than at other temperatures (Figure 4). Moreover, the lowest concentration of genomic DNA detected by the *B. abortus*-MCDA-LFB assay was 100 fg/μl when the reaction time was 30 min, while the LoDs were consistent (10 fg/μl) when the time range was from 40 to 60 min (Figure 5).

In the current study, the LoD of the *B. abortus*-MCDA LFB assay was 10 fg/μl (~3 copies/μl), which confirmed its excellent sensitivity for detecting genomic DNA of *B. abortus*. Notably, the LoD of the *B. abortus*-MCDA-LFB assay was 10-fold higher than that of *B. abortus*-LAMP-LFIA and 100-fold higher than that of *B. abortus*-PCR assay (Alamian et al., 2019; Moeini-Zanjani et al., 2020; Figure 6**)**. The prominent advantage of ultra-sensitivity plays a crucial role in improving the detectable rate, especially in cattle with a low bacterial load. In addition, the *B. abortus*-MCDA-LFB assay successfully identified all examined *B. abortus* strains (namely, reference strain, vaccine strain, and isolates) and did not cross-react with other *Brucella* spp. (including *Brucella melitensis*, *Brucella suis*, and *Brucella canis*) and non-*Brucella* pathogens, confirming that its specificity was 100% (Figure 7). Since the MCDA amplification requires only an isothermal condition (66°C, 40 min) and subsequent amplicons validation can be performed at room temperature using an AuNPs-LFB biosensor, the *B. abortus*-MCDA-LFB assay is ideally suited for identifying *B. abortus* in the field and/or resource-limited laboratories. Admittedly, the isothermal-based detection techniques, including LAMP and MCDA, are prone to aerosol contamination (Yang et al., 2021); therefore, the experimental steps (including mixture premixing, amplification, and AuNPs-LFB validation) must be performed in different laboratory zones.

In order to evaluate the applicability of *B. abortus*-MCDA-LFB assay, 56 whole blood samples were tested using conventional culture, *B. abortus*-PCR, *B. abortus*-LAMP-LFIA, and *B. abortus*-MCDA-LFB tests. In the detection of whole blood specimens, the sensitivity of the *B. abortus*-MCDA-LFB assay was consistent with that of the traditional culture method and *B. abortus*-LAMP test, which proved the reliability of the *B. abortus*-MCDA-LFB assay developed in the current study (Table 3; Supplementary Figure S1). Notably, the sensitivity of *B. abortus*-MCDA-LFB was higher than that of the *B. abortus*-PCR assay, which detected only seven whole blood samples (77.78%, 7/9), probably because the concentration of genomic DNA in the samples did not meet the limit of detection of *B. abortus*-PCR (Table 3; Supplementary Figure S1). Although the traditional culture is usually used as a standard method, it is difficult to be performed as a rapid screening/diagnostic tool due to its time-consuming, complex detection process, and especially the risk of infection for laboratory personnel. In addition, the detection workflow of the *B. abortus*-MCDA-LFB assay for whole blood samples can be completed within 70 min, including DNA template preparation (25 min), MCDA amplification (40 min), and AuNPs-LFB biosensor validation (2–5 min). Meanwhile, the cost of a single *B. abortus*-MCDA-LFB reaction is approximately 5.0 USD, including the MCDA amplification reagents (approximately 1.5 USD), the AuNPs-LFB biosensor (approximately 2.5 USD), and other reagents and materials (approximately 1.0 USD).

## Conclusion

The novel MCDA technique combined with a gold nanoparticle-based LFB biosensor targeting the *BruAb2_0168* gene (termed *B. abortus*-MCDA-LFB) was successfully established and evaluated in the current study. The *B. abortus*-MCDA-LFB assay, as a visual, rapid, readily available, and easy-to-use method, exhibited excellent sensitivity and high specificity for the identification of *B. abortus* strains in pure cultures or whole blood samples. Importantly, the AuNPs-LFB biosensor devised in our study can intuitively and accurately reflect the detection results, simplifying the test procedure and shortening the detection time. Taken together, the *B. abortus*-MCDA-LFB assay established in this study is a visual, fast, ultrasensitive, low-cost, easy-to-operate, and highly specific detection method, which can be used as a potential rapid identification tool for *B. abortus* infection in the field and/or resource-limited laboratories.

## Data availability statement

The original contributions presented in the study are included in the article/Supplementary material, further inquiries can be directed to the corresponding author.

## Author contributions

XinY and SL conceived and designed this study. SL supervised the study and revised the manuscript. XinY, SL, YW, YL, JH, XW, QT, XZ, and XiaY conducted the experiments. XinY, SL, YW, and YL analyzed the data. SL, YW, YL, and JH contributed the reagents and analysis tools. SL, YL, YW, and JH contributed the materials. XinY performed the software and drafted the manuscript. All authors contributed to the article and approved the submitted version.

## Funding

This study was funded by grants from National Natural Science Foundation of China (82273758), Science and Technology Department of Guizhou Province (Qiankehe platform talent [2018]5627).

## Conflict of interest

The authors declare that the research was conducted in the absence of any commercial or financial relationships that could be construed as a potential conflict of interest.

## Publisher’s note

All claims expressed in this article are solely those of the authors and do not necessarily represent those of their affiliated organizations, or those of the publisher, the editors and the reviewers. Any product that may be evaluated in this article, or claim that may be made by its manufacturer, is not guaranteed or endorsed by the publisher.

## Supplementary material

The Supplementary material for this article can be found online at: https://www.frontiersin.org/articles/10.3389/fmicb.2022.1071928/full#supplementary-material

## Abbreviations

*B. abortus*: *Brucella abortus*
MCDA: multiple cross displacement amplification
AuNPs-LFB: gold nanoparticles-based lateral flow biosensor
PCR: polymerase chain reaction
ATCC: American type culture collection
LoD: limit of detection
MG: malachite green
GZCDC: Guizhou Provincial Center for Disease Control and Prevention
FAM: carboxyfluorescein
nt: nucleotide
CL: control line
TL: test line

## References

[ref1] Alamian S. Zahraei Salehi T. Aghaiypour Kolyani K. Esmaelizad M. Etemadi A. (2019). Development of new modified simple polymerase chain reaction and real-time polymerase chain reaction for the identification of Iranian *Brucella abortus* strains. Arch. Razi Inst. 74, 235–241. doi: 10.22092/ARI.2018.122128.1218, PMID: 31592588

[ref2] Aliyev J. Alakbarova M. Garayusifova A. Omarov A. Aliyeva S. Fretin D.. (2022). Identification and molecular characterization of *Brucella abortus* and *Brucella melitensis* isolated from milk in cattle in Azerbaijan. BMC Vet. Res. 18, 71–79. doi: 10.1186/s12917-022-03155-1, PMID: 35168621 PMC8845251

[ref3] Ashmi M. Kumar B. Agrawal R. K. Prakash C. Abhishek Singh K. P. (2021). Development of BruAb2_0168 based isothermal polymerase spiral reaction assay for specific detection of Brucella abortus in clinical samples. Mol. Cell. Probes 59:101761. doi: 10.1016/j.mcp.2021.101761, PMID: 34400303

[ref4] Barua A. Kumar A. Thavaselvam D. Mangalgi S. Prakash A. Tiwari S.. (2016). Isolation & characterization of brucella melitensis isolated from patients suspected for human brucellosis in India. Indian J. Med. Res. 143, 652–658. doi: 10.4103/0971-5916.187115, PMID: 27488010 PMC4989840

[ref5] Batinga M. C. A. de Lima J. T. R. Gregori F. Diniz J. A. Muner K. Oliveira T. M. F. S.. (2018). Comparative application of IS711-based polymerase chain reaction (PCR) and loop-mediated isothermal amplification (LAMP) for canine brucellosis diagnosis. Mol. Cell. Probes 39, 1–6. doi: 10.1016/j.mcp.2018.02.003, PMID: 29524641

[ref6] Boggiatto P. M. Schaut R. G. Kanipe C. Kelly S. M. Narasimhan B. Jones D. E.. (2019). Sustained antigen release polyanhydride-based vaccine platform for immunization against bovine brucellosis. Heliyon 5:e02370. doi: 10.1016/j.heliyon.2019.e02370, PMID: 31517098 PMC6728543

[ref7] Chakravorty S. Simmons A. M. Rowneki M. Parmar H. Cao Y. Ryan J.. (2017). The new Xpert MTB/RIF ultra: improving detection of mycobacterium tuberculosis and resistance to rifampin in an assay suitable for point-of-care testing. MBio 8, 1–12. doi: 10.1128/mBio.00812-17, PMID: 28851844 PMC5574709

[ref8] Eltawab A. A. El-Hofy F. Hamdy M. Moustafa S. Soliman E. Ahmed W.. (2020). Isolation and molecular identification of brucella spp. in bovine herds kept at householders in the delta region of Egypt by maldi-tof and Amos-pcr. Vet. Ital. 56, 297–300. doi: 10.12834/VetIt.1980.10596.3, PMID: 33635618

[ref9] Gomes Y. Caterino-De-Araujo A. Campos K. Gonçalves M. G. Leite A. C. Lima M. A.. (2020). Loop-mediated isothermal amplification (LAMP) assay for rapid and accurate confirmatory diagnosis of HTLV-1/2 infection. Viruses 12, 1–15. doi: 10.3390/v12090981, PMID: 32899621 PMC7552020

[ref10] Gumaa M. M. Li Z. Cao X. Zhang N. Lou Z. Zhou J.. (2020). Specific detection and differentiation between Brucella melitensis and Brucella abortus by a duplex recombinase polymerase amplification assay. Front. Vet. Sci. 7, 1–13. doi: 10.3389/fvets.2020.539679, PMID: 33330681 PMC7732630

[ref11] Hinić V. Brodard I. Thomann A. Cvetnić Ž. Makaya P. V. Frey J.. (2008). Novel identification and differentiation of Brucella melitensis, B. abortus, B. suis, B. ovis, B. canis, and B. neotomae suitable for both conventional and real-time PCR systems. J. Microbiol. Methods 75, 375–378. doi: 10.1016/j.mimet.2008.07.002, PMID: 18675856

[ref12] Kang S. I. Her M. Kim J. W. Kim J. Y. Ko K. Y. Ha Y. M.. (2011). Advanced multiplex PCR assay for differentiation of Brucella species. Appl. Environ. Microbiol. 77, 6726–6728. doi: 10.1128/AEM.00581-11, PMID: 21666028 PMC3187128

[ref13] Kang S. I. Her M. Kim J. Y. Lee J. J. Lee K. Sung S. R.. (2015). Rapid and specific identification of Brucella abortus using the loop-mediated isothermal amplification (LAMP) assay. Comp. Immunol. Microbiol. Infect. Dis. 40, 1–6. doi: 10.1016/j.cimid.2015.03.001, PMID: 25841288

[ref14] Karthik K. Rathore R. Thomas P. Arun T. R. Viswas K. N. Agarwal R. K.. (2014). Loop-mediated isothermal amplification (LAMP) test for specific and rapid detection of Brucella abortus in cattle. Vet. Q. 34, 174–179. doi: 10.1080/01652176.2014.966172, PMID: 25220872

[ref15] Li S. Jiang W. Huang J. Liu Y. Ren L. Zhuang L.. (2020). Highly sensitive and specific diagnosis of coronavirus disease 19 (COVID-19) by reverse transcription multiple cross displacement amplification-labelled nanoparticles biosensor. Eur. Respir. J. 56:2002060. doi: 10.1183/13993003.02060-2020, PMID: 32859676 PMC7453731

[ref16] Li S. Liu Y. Chen X. Wang M. Hu W. Yan J. (2019a). Visual and rapid detection of *Leptospira interrogans* using multiple cross-displacement amplification coupled with nanoparticle-based lateral flow biosensor. Vector-Borne Zoonotic Dis. 19, 604–612. doi: 10.1089/vbz.2018.2395, PMID: 30702382

[ref17] Li S. Liu Y. Wang Y. Chen H. Liu C. Wang Y. (2019b). Lateral flow biosensor combined with loop-mediated isothermal amplification for simple, rapid, sensitive, and reliable detection of *Brucella* spp. Infect. Drug Resist. 12, 2343–2353. doi: 10.2147/IDR.S211644, PMID: 31440069 PMC6679679

[ref18] Li S. Liu Y. Wang Y. Wang M. Liu C. Wang Y. (2019c). Rapid detection of brucellaspp. and elimination of carryover using multiple cross displacement amplification coupled with nanoparticles-based lateral flow biosensor. Front. Cell. Infect. Microbiol. 9, 1–11. doi: 10.3389/fcimb.2019.00078, PMID: 30984627 PMC6447675

[ref19] Li F. Xiao J. Yang H. Yao Y. Li J. Zheng H.. (2022). Development of a rapid and efficient RPA-CRISPR/Cas12a assay for mycoplasma pneumoniae detection. Front. Microbiol. 13, 1–10. doi: 10.3389/fmicb.2022.858806, PMID: 35369478 PMC8965353

[ref20] López-Goñi I. García-Yoldi D. Marín C. M. De Miguel M. J. Muñoz P. M. Blasco J. M.. (2008). Evaluation of a multiplex PCR assay (Bruce-ladder) for molecular typing of all Brucella species, including the vaccine strains. J. Clin. Microbiol. 46, 3484–3487. doi: 10.1128/JCM.00837-08, PMID: 18716225 PMC2566117

[ref21] Moeini-Zanjani A. Pournajaf A. Ferdosi-Shahandashti E. Gholami M. Masjedian F. Khafri S.. (2020). Comparison of loop-mediated isothermal amplification and conventional PCR tests for diagnosis of common Brucella species. BMC. Res. Notes 13, 533–512. doi: 10.1186/s13104-020-05377-8, PMID: 33187548 PMC7666441

[ref22] Notomi T. Okayama H. Masubuchi H. Yonekawa T. Watanabe K. Amino N.. (2000). Loop-mediated isothermal amplification of DNA. Nucleic Acids Res. 28, 63e–663e. doi: 10.1093/NAR/28.12.E63, PMID: 10871386 PMC102748

[ref23] Paul S. Peddayelachagiri B. V. Gogoi M. Nagaraj S. Ramlal S. Konduru B.. (2020). Genome-wide unique insertion sequences among five Brucella species and demonstration of differential identification of Brucella by multiplex PCR assay. Sci. Rep. 10, 6368–6311. doi: 10.1038/s41598-020-62472-3, PMID: 32286356 PMC7156498

[ref24] Probert W. S. Schrader K. N. Khuong N. Y. Bystrom S. L. Graves M. H. (2004). Real-time multiplex PCR assay for detection of Brucella spp., B. abortus, and B. melitensis. J. Clin. Microbiol. 42, 1290–1293. doi: 10.1128/JCM.42.3.1290-1293.2004, PMID: 15004098 PMC356861

[ref25] Rahdar H. A. Golmohammadi R. Mirnejad R. Ataee R. A. Alishiri G. H. Kazemian H. (2018). Diversity of virulence genes in Brucella melitensis and Brucella abortus detected from patients with rheumatoid arthritis. Microb. Pathog. 118, 247–250. doi: 10.1016/j.micpath.2018.03.034, PMID: 29578063

[ref26] Rees R. K. Graves M. Caton N. Ely J. M. Probert W. S. (2009). Single tube identification and strain typing of Brucella melitensis by multiplex PCR. J. Microbiol. Methods 78, 66–70. doi: 10.1016/j.mimet.2009.04.010, PMID: 19410609

[ref27] Sergueev K. V. Filippov A. A. Nikolich M. P. (2017). Highly sensitive bacteriophage-based detection of Brucella abortus in mixed culture and spiked blood. Viruses 9:144. doi: 10.3390/v9060144, PMID: 28604602 PMC5490821

[ref28] Surucuoglu S. El S. Ural S. Gazi H. Kurutepe S. Taskiran P.. (2009). Evaluation of real-time PCR method for rapid diagnosis of brucellosis with different clinical manifestations. Polish J. Microbiol. 58, 15–19. 19469281

[ref29] Tekle M. Legesse M. Edao B. M. Ameni G. Mamo G. (2019). Isolation and identification of *Brucella melitensis* using bacteriological and molecular tools from aborted goats in the Afar region of North-Eastern Ethiopia. BMC Microbiol. 19, 108–106. doi: 10.1186/s12866-019-1474-y, PMID: 31126230 PMC6534919

[ref30] Wang Y. Li H. Li D. Li K. Wang Y. Xu J.. (2016a). Multiple cross displacement amplification combined with gold nanoparticle-based lateral flow biosensor for detection of *Vibrio parahaemolyticus*. Front. Microbiol. 7, 1–9. doi: 10.3389/fmicb.2016.02047, PMID: 28066368 PMC5177632

[ref31] Wang Y. Li H. Wang Y. Li H. Luo L. Xu J.. (2017). Development of multiple cross displacement amplification label-based gold nanoparticles lateral flow biosensor for detection of listeria monocytogenes. Int. J. Nanomed. 12, 473–486. doi: 10.2147/IJN.S123625, PMID: 28138243 PMC5238772

[ref32] Wang Y. Li H. Wang Y. Xu H. Xu J. Ye C. (2018). Antarctic thermolabile uracil-DNA-glycosylase-supplemented multiple cross displacement amplification using a label-based nanoparticle lateral flow biosensor for the simultaneous detection of nucleic acid sequences and elimination of carryover contaminatio. Nano Res.. 2017 115 11, 2632–2647 11, 2632–2647. doi: 10.1007/S12274-017-1893-Z

[ref33] Wang Y. Wang Y. Ma A. J. Li D. X. Luo L. J. Liu D. X.. (2015). Rapid and sensitive isothermal detection of nucleic-acid sequence by multiple cross displacement amplification. Sci. Rep. 5, 1–16. doi: 10.1038/srep11902, PMID: 26154567 PMC4648395

[ref34] Wang Y. Wang Y. Xu J. Ye C. (2016b). Development of multiple cross displacement amplification label-based gold nanoparticles lateral flow biosensor for detection of Shigella spp. Front. Microbiol. 7, 1–13. doi: 10.3389/fmicb.2016.01834, PMID: 27917160 PMC5114309

[ref35] Wang Y. Zhao X. Cheng J. Tang X. Chen X. Yu H.. (2021). Development and application of a multiple cross displacement amplification combined with nanoparticle-based lateral flow biosensor assay to detect Candida tropicalis. Front. Microbiol. 12, 1–10. doi: 10.3389/fmicb.2021.681488, PMID: 34177867 PMC8222920

[ref36] Yagupsky P. Morata P. Colmenero J. D. (2020). Laboratory diagnosis of human brucellosis. Clin. Microbiol. Rev. 33, 1–54. doi: 10.1128/CMR.00073-19 PMC6860005 31722888

[ref37] Yang X. Wang Y. Liu Y. Huang J. Tan Q. Ying X.. (2021). A label-based polymer nanoparticles biosensor combined with loop-mediated isothermal amplification for rapid, sensitive, and highly specific identification of *Brucella abortus*. Front. Bioeng. Biotehnol. 9, 1–12. doi: 10.3389/fbioe.2021.758564, PMID: 34869267 PMC8636779

