# Supplementary material for: Rapid, ultrasensitive, and highly specific identification of Brucella abortus utilizing multiple cross displacement amplification combined with a gold nanoparticles-based lateral flow biosensor
Source: Front Microbiol. 2022 Nov 29;13:1071928. doi: 10.3389/fmicb.2022.1071928 (PMC9744775; doi:10.3389/fmicb.2022.1071928)
Supplement: Supplementary file 1 [file Data_Sheet_1.docx]

Supplementary Material

**Rapid, ultrasensitive, and highly specific identification of *Brucella abortus* utilizing multiple cross displacement amplification combined with a nanoparticles-based lateral flow biosensor**

Xinggui Yang ^1^, Yue Wang ^1^, Ying Liu ^1^, Junfei Huang ^1^, Xiaoyu Wei ^1^, Qinqin Tan ^1^, Xiaoyan Zeng ^2^, Xia Ying ^1^, Shijun Li ^1 *^

^1^ Laboratory of Infectious Disease of Experimental Center, Guizhou Provincial Center for Disease Control and Prevention, Guiyang, 550004, Guizhou, P.R. China.

^2^ The Second Affiliated Hospital, Guizhou University of Traditional Chinese Medicine, Guiyang, 550003, Guizhou, P.R. China.

***Correspondence**

Shijun Li

Email: zjumedjun@163.com.

**Materials and Methods**

**The *B. abortus*-LAMP-LFIA assay**

The reaction mixtures (25 μl) of *B. abortus*-LAMP-LFIA contained the following: 12.5 μl reaction buffer (2 ×), 1 μl *Bst* DNA polymerase (8 U), 1.6 μM each of *Bru*-FIP* (5'-FAM-AACCCGACACAGCAAGCGTGGCAAGACGGCGCAGTT-3') and *Bru*-BIP (5'- ATAGTGGCAATACGACGATTGCGATTGCCCGCAAGCCTG-3'), 0.8 μM each of *Bru*-LF (5'-CGTCAGCTTGTTCGAG-3') and *Bru*-LB (5'-GATGGCAGCACGGAT-3'), 0.4 μM each of *Bru*-F3 (5'-CCATCACGATCGATGGCG-3') and *Bru*-B3 (5'-AGTGTGCCCGCATTGG-3'), 1 μl of biotin-14-dCTP, 1 μl MG indicator, 5 μl DNA templates extracted from whole blood samples, and nuclease-free water was added to 25 µl. Follow-up, the reaction tube was incubated at 65 °C for 50 min and then inactivated at 85 °C for 5 min.

**The *B. abortus*-PCR assay**

The amplification system (25 μl) included the following: 12.5 μl 2 × Taq Master Mix (CoWin Biosciences Co., Ltd. Beijing, China), 0.2 μM *Bru*-F (5’-TCGCATCGGCAGTTTCAA-3’), 0.2 μM *Bru*-R (5’-CCAGCTTTTGGCCTTTTCC-3’), 5 μl DNA templates extracted from whole blood specimens, and nuclease-free water was added to 25 μl. The mixture tubes were performed by an automated thermal cycler (Hangzhou Bori Technology Co., Ltd. Hangzhou, China). The reaction systems were denatured at 94 °C for 2 min, and 35 reaction cycles were conducted. The cycles consisted of denaturation at 94 °C (30 s), annealing at 59 °C (30 s), and primer extension at 72 °C (30 s). The final extension time was set for 2 min. The PCR amplicons were verified in a 1.5% agarose gel with GelRed staining under UV light (BioRad, USA).

**Supplementary Figure**


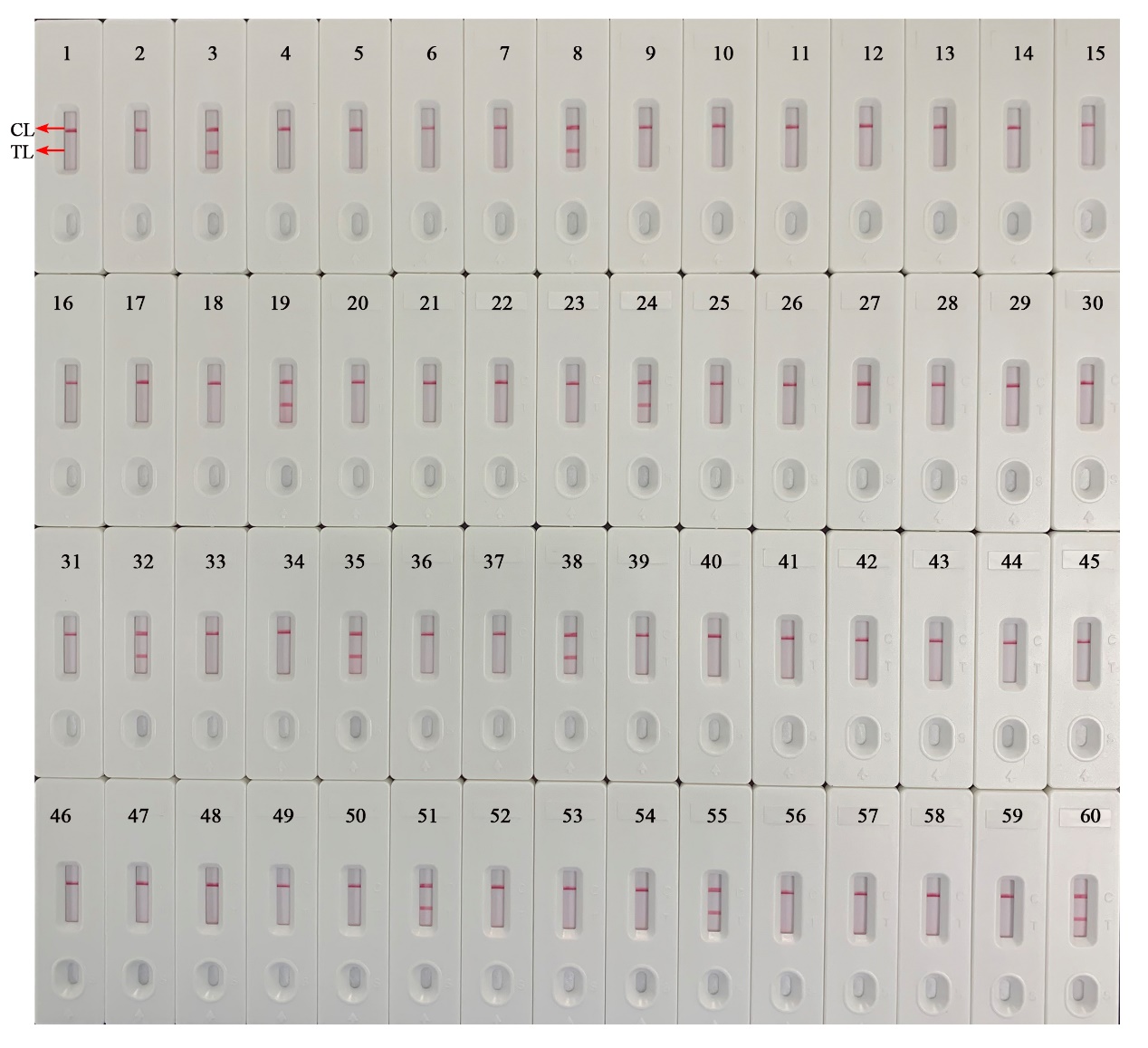


**Supplementary Figure S1.** Detection results of the *B. abortus*-MCDA-LFB assay for 56 whole blood samples. DNA templates extracted from 56 whole blood samples were examined to evaluate the applicability of the *B. abortus*-MCDA-LFB assay. Biosensors 1-56, the detection results of 56 whole blood samples. Biosensor 57, negative control (1 µl of genomic DNA from the *Mycobacterium tuberculosis* H37Rv), Biosensor 58, negative control (1 µl of liquid from the environmental sample in the test); Biosensor 59, blank control (nuclease-free water), Biosensor 60, positive control (1 µl of genomic DNA from the *B. abortus* strain). MCDA, multiple cross displacement amplification; AuNPs-LFB, gold nanoparticles-based lateral flow biosensor; TL, test line; CL, control line.
